# Using Twins to Better Understand Sibling Relationships

**DOI:** 10.1007/s10519-016-9825-z

**Published:** 2016-10-28

**Authors:** Katharine M. Mark, Alison Pike, Rachel M. Latham, Bonamy R. Oliver

**Affiliations:** School of Psychology, University of Sussex, Brighton, East Sussex BN1 9QH UK

**Keywords:** Twins, Siblings, Sibling relationship quality, Behaviour genetics

## Abstract

We compared the nature of the sibling relationship in dyads of varying genetic relatedness, employing a behavioural genetic design to estimate the contribution that genes and the environment have on this familial bond. Two samples were used—the Sisters and Brothers Study consisted of 173 families with two target non-twin children (mean ages = 7.42 and 5.22 years respectively); and the Twins, Family and Behaviour study included 234 families with two target twin children (mean age = 4.70 years). Mothers and fathers reported on their children’s relationship with each other, via a postal questionnaire (the Sisters and Brothers Study) or a telephone interview (the Twins, Family and Behaviour study). Contrary to expectations, no mean level differences emerged when monozygotic twin pairs, dizygotic twin pairs, and non-twin pairs were compared on their sibling relationship quality. Behavioural genetic analyses also revealed that the sibling bond was modestly to moderately influenced by the genetic propensities of the children within the dyad, and moderately to substantially influenced by the shared environment common to both siblings. In addition, for sibling negativity, we found evidence of twin-specific environmental influence—dizygotic twins showed more reciprocity than did non-twins. Our findings have repercussions for the broader application of results from future twin-based investigations.

## Introduction

Siblings (and twins specifically) have played a prominent role in genetically sensitive studies. For example, pairs of siblings of varying genetic relatedness (i.e., monozygotic (MZ, or identical) and dizygotic (DZ, or fraternal) twins) have been used to understand the genetic and environmental contributions for specific traits (McGuire, 2001). However, similarities and differences between siblings have dominated this literature, whilst sibling relationship quality (SRQ) in its own right has been relatively neglected (McGuire et al. 2015). The aim of the current study was to focus on the nature of sibling dynamics from a behavioural genetic (BG) perspective. We compared levels of positivity and negativity within the sibling relationship for differing sibling pairs (MZ twins, DZ twins, and non-twin siblings). In addition, we estimated the contribution that genes, the shared environment and the non-shared environment make to this phenotype. We investigated these goals using parental reports of sibling relationships, with two samples (one twin sample and one non-twin sample) of brothers and sisters in early to middle childhood.

### SRQ

Much research attention has been given to the nature of the relationship between non-twin brothers and sisters in recent decades (Brody 1998; McHale et al. 2012). For many, sibling relationships are their most enduring, starting in infancy and persisting through to old age (Cicirelli, 1982). During childhood specifically, siblings spend much of their time together, often more than with parents or peers (McHale and Crouter 1996), and these intense relationships are typically characterised by both spontaneity and ambivalence (Dunn and Kendrick 1982). The quality of the sibling relationship in these early years has been linked with social adjustment and well-being throughout the life span (Brody 2004). Both cooperative and affectionate behaviours, as well as conflictual and hostile behaviours, within these interactions play an important role in children’s development (Furman and Buhrmester 1992).

Gender influences on SRQ are robust. In general, girls show more positive behaviour towards siblings than do boys (Abramovitch et al. 1986), with older sisters being particularly prosocial towards their younger siblings (Abramovitch et al. 1979). Contrastingly, boys have been found to engage in more negative sibling behaviours, such as physical aggression, arguing, and teasing (Brody et al. 1985). Dyadic gender differences also follow this pattern. For example, Buist et al. (2002) showed that sisters have a significantly greater attachment to each other than do brothers, and Maccoby (1998) argued that pairs of brothers display particularly high levels of antisocial behaviour.

### SRQ in twin dyads

Despite the wealth of studies that have focused on SRQ and its correlates and consequences, few have targeted the twin relationship. These same-age individuals represent an unusual type of sibling dyad, although data from the Office for National Statistics (ONS; 2013) suggest that they are becoming increasingly common-15.6 out of every 1000 deliveries were multiple births in the year 2013. Alongside serving a valuable role in genetically sensitive designs (Plomin et al. 2013), twins have captivated the public’s imagination, perhaps because both classic literature and the modern media portray this type of sibling bond as one that is exceptionally special and intimate (Burlington 1945; Playfair 2002; Segal 1999).

Twin relationship research has drawn on the theoretical perspectives of kin selection and inclusive fitness. These outlooks emphasise natural selection, whereby individuals attempt to ensure the survival of their own genes by protecting closely related family members over all others (Hamilton 1964). In line with such a concept, and according to Neyer (2002), MZ twins have a special regard for one another because they share more of their genetic makeup than do DZ twins or non-twin siblings. Thus, they may be more motivated to behave altruistically towards each other, to invest in their co-twin’s well-being, and to rely on each other, in order to guarantee their (and their twin’s) reproductive success (Neyer and Lang 2003). Indeed, research following a large Finnish cohort of teenage twins revealed that MZ pairs were more likely to report being dependent on their twin sibling than were DZ pairs (Penninkilampi-Kerola et al. 2005), a result that was replicated using maternal reports in three-year-old twin pairs as well (Fortuna et al. 2010). Similarly, according to Scarr and McCartney (1983), children (and adults) belonging to a MZ dyad generally choose each other as friends and companions to a greater extent than do individuals belonging to a DZ dyad.

Using the alternative attachment-theoretical explanation, Tancredy and Fraley (2006) have argued that the so-called ‘twin situation’ naturally encourages the development of a secure attachment bond, regardless of whether the target siblings are MZ or DZ twins. These authors claim that twins form distinctively close relationships in comparison to non-twin siblings, due to circumstances such as sharing birthdays, peer groups, and bedrooms, and spending a lot of time in proximity to one another. Further support for attachment theory comes from Fraley and Tancredy’s (2012) later work, which suggests that twin children rely more heavily on their co-twin for safety and security than do non-twin siblings. Differently-aged siblings also claim to be happier without their brothers or sisters around, whereas twins state a preference for being in each other’s company (Segal 1999). This derivative of attachment theory therefore places less of an emphasis on genetic relatedness, instead highlighting the importance of the distinct environment that twins experience.

There are marked divergences in the main theoretical studies discussed (i.e., Fraley and Tancredy 2012; Neyer 2002; Neyer and Lang 2003; Tancredy and Fraley 2006), in terms of the age and gender of the participants used, as well as the methods employed. For example, Neyer et al., who argue for an inclusive fitness interpretation, explored twin relationships in old age, whereas Fraley and Tancredy, who support an attachment framework, recruited younger adults. Neyer himself acknowledges that the bond between siblings changes across development, thus differently-aged samples may well have influenced the dissimilar trajectories put forward by the two theories. Similarly, the gender of the children included varied across the four central studies—same-sex twin pairs were used for the inclusive fitness research (Neyer et al.), whereas a group of mixed-sex siblings were tested in the attachment research (Tancredy and Fraley). Including opposite-sex DZ and non-twin dyads in Neyer’s papers may have resulted in differences emerging between identical and fraternal pairs’ interactions, as predicted by attachment-based theories. Finally, Neyer et al. carried out detailed interviews with their twins to capture the nature of the sibling relationship, whereas Fraley and Tancredy used a one-item online questionnaire to assess SRQ. It seems likely that the rather broad latter measure would fail to differentiate between the groups of siblings, and this should be borne in mind.

### BG and SRQ

BG is a field of study in which phenotypic variation among individuals is separated into heritable and environmental components, using family members (often siblings) of differing genetic relatedness (Plomin et al. 1977). For example, MZ and DZ pairs’ intraclass correlations are compared, and significant heritability is assumed if these values are considerably higher for MZ than for DZ twins. SRQ dimensions can themselves be treated as phenotypes, to which BG techniques can be applied.

There are few studies that have examined genetic and environmental contributions to twin SRQ (McGuire et al. 2015), and those that are available have varied in terms of the age of the participants, ranging from young childhood (Lemery and Goldsmith 2001) through to mid-adolescence (Pike and Atzaba-Poria 2003); the size of the sample, ranging from 124 children (Rende et al. 1992) through to 701 (Plomin 1994); and the measures employed, ranging from parental questionnaires (Lemery and Goldsmith) through to unstructured observations (Rende et al.). Only two such studies, those conducted by Lemery and Goldsmith and Pike and Atzaba-Poria, have made twin SRQ their focus, with the remainder concentrating on adoptive and non-adoptive siblings pairs (e.g., Rende et al.). Impressively, Reiss et al. (2000) report findings regarding adolescent SRQ from six family types, incorporating a twin and step-family design within a single study. Despite the vast variations across the relevant research, the results of these studies have been broadly similar, allowing researchers to glean insights into the ways in which genes and the environment influence SRQ.

BG has demonstrated evidence of a modest genetic contribution to SRQ, yet the extent to which this heritability influences positivity (characterised by warmth, closeness, and affection; Furman and Buhrmester 1985) and negativity (characterised by aggression, competition, and rivalry; Furman and Buhrmester 1985) between siblings varies from study to study. Interestingly, Lemery and Goldsmith (2001) discovered that there was negligible (and non-significant) genetic influence on their measure of sibling cooperation, whilst a substantial heritability estimate (of 41 %) emerged for sibling conflict. Pike and Atzaba-Poria (2003) also replicated this pattern of results, finding that sibling rivalry and hostility were strongly affected by genes, but that sibling affection was not. Generally, it has been found that aggressive behaviour is influenced by genes to a greater extent than is prosocial behaviour (Eley et al. 1999), and this outcome also plays out in the parenting literature (Oliver et al. 2014). However, some findings suggest otherwise-for example Rende et al. (1992) uncovered more substantial genetic influence for sibling positivity and cooperation than for sibling negativity and conflict.

As well as genetic factors, shared environmental influence has also emerged as an important contributor to SRQ (McGuire et al. 2015; Reiss et al. 2000). For Lemery and Goldsmith (2001), these estimates accounted for 6 % of variance in sibling positivity, and 28 % in sibling negativity. Correspondingly, large and significant shared environment values were found by Rende et al. (1992). For example, sibling cooperation yielded an estimate of 75 %, with this increasing to 85 % for sibling conflict. McGuire et al. (2000) focused their paper on sibling negativity, using a sample of full (biological) siblings and unrelated (adoptive) siblings, rather than twins. The authors reported evidence of a significant environmental contribution towards conflict within these dyads when children’s reports were explored. All of these findings indicate that siblings tend to behave in a reciprocal way towards one another, perhaps due to the general family environment, or specific parenting styles, that both children experience within the home (Pike and Atzaba-Poria 2003).

### The present study

Much of the existing research exploring SRQ in twins has compared MZ and DZ twins in the absence of a non-twin group (e.g., Penninkilampi-Kerola et al. 2005), or compared twins to non-twins, without considering twin zygosity (e.g., Tancredy and Fraley 2006). With the exception of Reiss et al.’s (2000) work, the BG papers discussed here have also either left out non-twin siblings in their research (e.g., Lemery and Goldsmith 2001), or have recruited biological siblings versus adoptive siblings (e.g., McGuire et al. 2000). These methodologies make it difficult to confirm, or disconfirm, inclusive fitness or attachment theories when studying sibling relationships. For the first time, we compared the nature of the relationship between MZ twins, DZ twins, and non-twin siblings in early to middle childhood, and, within the same study, we used BG techniques to disentangle genetic and environmental contributions to SRQ.

Using parental reports of SRQ, we tested the rival hypotheses that either (a) MZ twins would have higher levels of SRQ positivity, and lower levels of SRQ negativity, than DZ twins or non-twin siblings, in line with Neyer and Lang’s (2003) interpretation of inclusive fitness theory; or that (b) MZ twins and DZ twins would have higher levels of SRQ positivity, and lower levels of SRQ negativity, than non-twin siblings, in line with Tancredy and Fraley’s (2006) secure attachment explanation. It was also hypothesised that (c) SRQ would differ significantly as a function of gender. As suggested by previous research (Buist et al. 2002; Maccoby 1998), it was expected that female-female sibling dyads would have higher levels of SRQ positivity than male-male or opposite-sex dyads, and that male-male sibling dyads would have higher levels of SRQ negativity than female-female or opposite-sex dyads. Finally, using a BG approach, it was hypothesised that (d) SRQ positivity would yield substantial shared environmental influence and modest genetic influence; and that (e) SRQ negativity would yield substantial genetic influence and modest shared environmental influence.

## Method

### Participants and recruitment

The study used two samples. The first consisted of 173 families, each with two non-twin children, from the Sisters and Brothers Study (SiBS; Pike et al. 2006). Schools in the south of England were approached and asked to send letters to parents of children in reception (4- to 5-year-olds) and Year 1 (5- to 6-year-olds) classes who had an older brother or sister aged 8 years or younger. However, many were unable (or unwilling) to target specific children and sent letters to all children in these classes. Letters were sent home via the children, although there was no guarantee that parents received these. Because of this opt-in procedure, it was not possible to estimate refusal rates accurately.

Within this sample, 118 families (68.2 %) were two-parent families and 55 (31.8 %) were single-mother families. Both mothers and fathers participated in 101 of the families (58.1 %). In 68 of the remaining families, data were collected from mothers only, and for the additional two families, data were collected from fathers only. We did not restrict our inclusion criteria to two-parent families, because we were interested in obtaining views from as many parents as possible. The average ages of the mothers and fathers were 36.20 years (*SD* = 4.99) and 40.31 years (*SD* = 5.18) respectively. For the older siblings and younger siblings, the average ages were 7.42 years (*SD* = 0.84) and 5.22 years (*SD* = 0.61) respectively. Parents ranged from working- to middle-class in terms of their educational and occupational backgrounds, and approximately equal numbers of the four sibling sex constellations (boy–boy, boy–girl, girl–girl, and girl–boy) were present in the sample.

The second sample saw data collected from mothers and fathers, along with their twin children, as part of the Twins, Family and Behaviour (TFaB) longitudinal study. Mothers of twins born in England or Wales in 2009 were contacted by the ONS and asked if they would like to participate in the study. Following this, we contacted mothers to ask whether they had a partner living with them who would be willing to take part. In addition, we expanded our sample by: (a) broadening the participation criteria to include twins born in 2010 as well as in 2009; (b) asking participants to put us in touch with any other families they knew who might like to take part; and (c) advertising through online social media Twitter tweets from a well-known registered UK twins charity [the Twins and Multiple Births Association (TAMBA)]. The ONS approached 800 mothers, and 287 (35.9 %) agreed to take part by returning a form detailing their contact information. Of these 287 mothers, 274 returned their initial questionnaire. One hundred and twenty-three fathers (121 biological fathers, 1 stepfather and 1 guardian of the twins) also agreed to be involved, and a further 59 families came forward to participate following our additional recruitment attempts. Thus 346 families were recruited overall, of which 274 were consistently engaged with the research.

This paper focused on information obtained from telephone interviews conducted with 234 of these families of twins. Both mothers and fathers participated in 103 of the families (44.0 %). In 127 of the remaining families, data were collected from mothers only, and for the additional four families, data were collected from fathers only. Table 1 shows demographic information for the participations.

**Table 1:** Demographic information for the sample

Demographic information	Mother-specific		Father-specific		Twin-specific	
	*N* = 230	%	*N* = 107	%	*N* = 234	%
Marital status						
Married to parent of twins	182	79.1	–	–	–	–
Cohabiting with parent of twins	20	8.7				
Married to other	2	0.9				
Cohabiting with other	4	1.7				
Single unmarried	11	4.8				
Single divorced	5	2.2				
Single separated	4	1.7				
Single widowed	2	0.9				
Highest educational qualification						
Post-graduate degree	72	31.3	24	25.0	–	–
Undergraduate degree	74	32.2	34	35.4		
2 + A level passes (grades A–E)	19	8.3	9	9.4		
1 A level pass (grades A–E)	7	3.0	6	6.3		
5 + GCSEs or O levels (grades A–C)	18	7.8	6	6.3		
1–4 GCSEs or O levels (grades A–C)	20	8.7	11	11.5		
GCSE(s) or O level(s) with grades D–G	12	5.2	4	4.2		
Other qualifications obtained outside the UK	6	2.6	2	2.1		
No qualifications	2	0.9	0	0		
Twin zygosity						
MZ pairs	–	–	–	–	84	36.5
DZ same-sex pairs					76	33.0
DZ opposite-sex pairs					70	30.4
Unclassified					4	1.71
Age	*M* = 38.78		*M* = 40.89		*M* = 4.70	
	*SD* = 4.45		*SD* = 6.41		*SD* = 0.37	

*MZ* monozygotic/identical twins, *DZ* dizygotic/fraternal twins, *N* 96 for father reports of highest educational qualifications, as 11 did not answer this question

### Procedure and measures

As part of the SiBS (Pike et al. 2006), participating families were visited at home, where parents and children were interviewed and parents completed questionnaires. The TFaB study did not include any home visits. Instead, involved parents were asked to complete a postal questionnaire and a 40-minute telephone interview.

#### Parent report of sibling zygosity

We classified twins within the TFaB study as either MZ or DZ, via a parent report zygosity questionnaire designed by Price et al. (2000) and adapted from Goldsmith’s (1991) original scale. The measure involves two steps for classifying zygosity, and has been found to be highly reliable in comparison to blood (Plomin et al. 1991) and DNA (Price et al. 2000) testing procedures. Firstly, certain individual items are used as definite markers of zygosity. Twins described as looking as alike as ‘two peas in a pod’ by their parents, as opposed to looking as alike as ‘brothers and sisters’ or not looking ‘much alike at all’, were classified as MZ. This question alone has been shown to correctly categorise a high proportion of twin pairs (Cederlof et al. 1961). Twins described as not looking ‘much alike at all’ or as having ‘clear differences’ in eye colour, hair colour or hair texture were classified as DZ, except where they were described as being as alike as ‘two peas in a pod’, in which case they were left as unclassified. 83.1 % of same-sex twins were classified using these specific individual items. For the remaining twins, the items were scored numerically, with low scores given to responses indicating similarity between twins and high scores given to responses indicating dissimilarity between twins. For example, other questions asked were ‘Do any of the following people ever mistake the twins for each other? Other parent; older brothers or sisters; other relatives; babysitter/day carer; parents’ close friends; parents’ casual friends; people meeting the twins for the first time’. Answers to these questions were rated on a four-point scale, where 1 = yes, often and 4 = never/rarely. Parents were also asked whether the twins’ teeth began to come through at the same time, and whether they can tell the twins apart when looking at a new photograph. The scores for questions that were answered were summed and then divided by a maximum possible score, in order to create a physical similarly quotient (PSQ) lying between 0 (representing maximum physical similarity) and 1 (indicating maximum physical dissimilarity). Twin pairs with PSQ scores below the median were classified as MZ, and twin pairs with PSQ scores above the median were classified as DZ.

#### Parent report of SRQ

Both twin and non-twin SRQ was measured using the same adapted version of the widely used Maternal Interview of Sibling Relationships (MISR; Stocker et al. 1989). In the SiBS, parents completed this via a questionnaire, and in the TFaB study, the same items were read aloud to parents during their telephone interview. Parents were asked to rate how often their children displayed 13 varying behaviours relating to different aspects of the sibling relationship, including companionship, quarrels, sharing, competing and jealousy. Four of the items were scored for the sibling relationship overall (for example, ‘Of the time the siblings spend together, how often do they play together?’), and nine required ratings for the older sibling/twin 1 and the younger sibling/twin 2 individually (for example, ‘On a day-to-day basis, how often does (sibling 1) show affection towards (sibling 2)?; and how often does (sibling 2) show affection towards (sibling 1)?’). Varying response scales were used throughout; the most commonly used were a percentage-based scale, where 1 = less than 5 % of their time together and 6 = almost all of their time together, and a frequency-based scale, where 1 = once a month or less and 6 = just about every day. Factor analysis yielded composite scores for SRQ positivity (11 items) and SRQ negativity (3 items). Resultant Cronbach’s alphas for these scales were 0.85 and 0.84 for SRQ positivity (for mother and father reports, respectively) and 0.78 and 0.74 for SRQ negativity (for mother and father reports, respectively).

## Results

### Preliminary analyses

We created unstandardised residual variables for mother and father reports of SRQ positivity and negativity, in order to control for the mean age of the sibling dyad. Table 2 shows descriptive statistics for these MISR variables, across the three sibling zygosity groups (MZ twin pairs, DZ twin pairs, and non-twin pairs). Table 3 presents similar descriptive statistics for the three sibling sex constellation groups (male-male pairs, female-female pairs, and opposite-sex pairs).

**Table 2:** Descriptive statistics for the MISR composite scales, as a function of sibling zygosity

MISR scales	MZ twin pairs		DZ twin pairs		Non-twin pairs	
	*M*	*SD*	*M*	*SD*	*M*	*SD*
Mother reports SRQ positivity	0.12	0.62	0.06	0.62	−0.11	0.71
Mother reports SRQ negativity	0.07	1.17	0.10	1.13	−0.12	1.00
Father reports SRQ positivity	0.16	0.58	0.01	0.61	−0.08	0.68
Father reports SRQ negativity	0.09	1.01	0.15	0.97	−0.09	0.95

The MISR composite scales used are unstandardised residuals that control for mean age of siblings

*MISR* Maternal Interview of Sibling Relationships, *SRQ* sibling relationship quality, *MZ* monozygotic/identical twins, *DZ* dizygotic/fraternal twins

**Table 3:** Descriptive statistics for the MISR composite scales, as a function of sibling sex constellation

MISR scales	Male-male pairs		Female-female pairs		Opposite-sex pairs	
	*M*	*SD*	*M*	*SD*	*M*	*SD*
Mother reports SRQ positivity	−0.08	0.76	0.14	0.56	−0.07	0.66
Mother reports SRQ negativity	0.31	1.12	−0.12	1.09	−0.12	1.04
Father reports SRQ positivity	−0.15	0.79	0.17	0.46	−0.02	0.60
Father reports SRQ negativity	0.22	0.92	−0.08	0.97	−0.11	1.02

The MISR composite scales used are unstandardised residuals that control for mean age of siblings

*MISR* maternal interview of sibling relationships, *SRQ* sibling relationship quality

Table 4 shows correlations among the MISR variables. Father reports of SRQ positivity and negativity were moderately correlated, but mother reports were not. Mother and father reports of SRQ positivity were highly correlated, as were their reports of negativity.

**Table 4:** Correlations among the MISR composite scales

MISR scales	1	2	3	4
Mother reports SRQ positivity				
Mother reports SRQ negativity	−0.06			
Father reports SRQ positivity	0.49**	−0.19**		
Father reports SRQ negativity	−0.13*	0.50**	−0.21**	

The MISR composite scales used are unstandardised residuals that control for mean age of siblings

*MISR* maternal interview of sibling relationships, *SRQ* sibling relationship quality

* *p*  < 0.05, ** *p* < 0.01

### Two-way analysis of variance (ANOVA) tests

In order to assess mean-level differences on the MISR, for both the sibling zygosity groups and the sibling sex constellation groups, we carried out four two-way (sibling zygosity x sibling sex constellation) ANOVAs, using mother reports of SRQ positivity, mother reports of SRQ negativity, father reports of SRQ positivity, and father reports of SRQ negativity as the dependent variables. Three of these tests had strong observed power (Field, 2013), with estimates above 0.8 (mother reports of SRQ positivity = 0.94; mother reports of SRQ negativity = 0.87; and father reports of SRQ positivity = 0.88). Father reports of SRQ negativity had a marginally lower power value of 0.75.

#### Sibling zygosity

Unexpectedly, there was a non-significant main effect of sibling zygosity on SRQ, for all of the four models tested: *F*(3, 389) = 1.61, *p* = 0.187 for mother reports of SRQ positivity; *F*(3, 389) = 0.86, *p* = 0.465 for mother reports of SRQ negativity; *F*(3, 200) = 1.73, *p* = 0.162 for father reports of SRQ positivity; and *F*(3, 200) = 1.06, *p* = 0.369 for father reports of SRQ negativity.

Neither our hypothesis (a), that MZ twins would have higher levels of SRQ positivity and lower levels of SRQ negativity than DZ twins or non-twin siblings; nor our hypothesis (b), that MZ twins and DZ twins would have higher levels of SRQ positivity and lower levels of SRQ negativity than non-twin siblings, were supported.

#### Sibling sex constellation

Unexpectedly, there was a non-significant main effect of sibling sex constellation on mother reports of SRQ positivity, *F*(2, 389) = 0.37, *p* = 0.690; father reports of SRQ positivity, *F*(2, 200) = 1.92, *p* = 0.149; and father reports of SRQ negativity, *F*(2, 200) = 1.47, *p* = 0.232. However, there was a significant main effect on mother reports of SRQ negativity, *F*(2, 389) = 3.41, *p* = 0.034. As recommended by Field (2013), we used Gabriel post hoc tests, due to the difference in sample sizes. These tests revealed significant differences between male-male pairs and female-female pairs (*p* = 0.006), with the former scoring more highly on SRQ negativity (*M* = 0.39) than the latter (*M* = −0.22). There was also a significant difference between male-male pairs and opposite-sex pairs (*p* = 0.005), again with the former scoring more highly on SRQ negativity (*M* = 0.31) than the latter (*M* = −0.10). There was a non-significant difference between female-female pairs and opposite-sex pairs for mother reports of SRQ negativity (*p* = 1.00).

These findings partially supported our hypothesis (c)-female-female sibling dyads did not have higher levels of SRQ positivity than male-male or opposite-sex dyads; however, male-male sibling dyads did show higher levels of SRQ negativity than female-female or opposite-sex dyads when mother reports were considered.

#### Interaction effects

There was a non-significant interaction effect between sibling zygosity and sibling sex constellation on SRQ: *F*(4, 389) = 1.36, *p* = 0.247 for mother reports of SRQ positivity; *F*(4, 389) = 0.58, *p* = 0.679 for mother reports of SRQ negativity; *F*(4, 200) = 1.22, *p* = 0.302 for father reports of SRQ positivity; and *F*(4, 200) = 1.45, *p* = 0.218 for father reports of SRQ negativity.

### BG analyses

For the remaining analyses, and in contrast to the dyadic SRQ positivity and negativity values used in the ANOVAs, we calculated two individual scores for the older sibling/twin 1 and the younger sibling/twin 2 for the SRQ constructs. This was done by creating a sibling 1 and a sibling 2 average across the nine MISR items that required ratings for the two children individually. We then created unstandardised residual variables, in order to control for the mean age of the sibling dyad, as well as for each child’s sex.

#### Intraclass correlations

Table 5 shows twin intraclass correlations. MZ twins had consistently higher correlations than both DZ twins and non-twin siblings, suggesting genetic influence. The correlations were also fairly large across the three groups, indicating shared environmental influence. Finally, the MZ correlations were very high overall, suggesting little non-shared environmental influence.

**Table 5:** Intraclass correlations among the MISR composite scales, as a function of sibling zygosity

MISR scales	Sibling intraclass correlations		
	MZ twin pairs	DZ twin pairs	Non-twin pairs
Mother reports SRQ positivity	0.89***	0.60***	0.69***
Mother reports SRQ negativity	0.93***	0.74***	0.65***
Father reports SRQ positivity	0.85***	0.80***	0.78***
Father reports SRQ negativity	0.97***	0.72***	0.73***

The MISR scales used are unstandardised residuals that control for mean age of siblings and sex of each child

*MISR* maternal interview of sibling relationships, *SRQ* sibling relationship quality, *MZ* monozygotic/identical twins, *DZ* dizygotic/fraternal twins

*** *p*  < 0.001

#### Univariate ACTE quantitative analyses

Although intraclass correlations are informative, a model fitting approach is a more powerful and explicit way of testing genetic and environmental contributions to SRQ (Eaves et al. 1978). This method also provides additional information, lacking in correlation tests, such as confidence intervals for variance estimates. In this study, maximum-likelihood model fitting analyses were performed using the program R (R Development Core Team 2009) and the modelling package Open Mx (Neale 1991). The ACTE BG model (Jinks and Fulker 1970) was assumed, which evaluates the effects of additive genetic influences (A), shared environmental influences (C), twin-specific environmental influences (T), and non-shared environmental influences (E). ‘T’ was incorporated here to test whether twins, being the same age, may be more similar to one another than would be expected based on genetic or shared environmental factors.

**Fig. 1:**
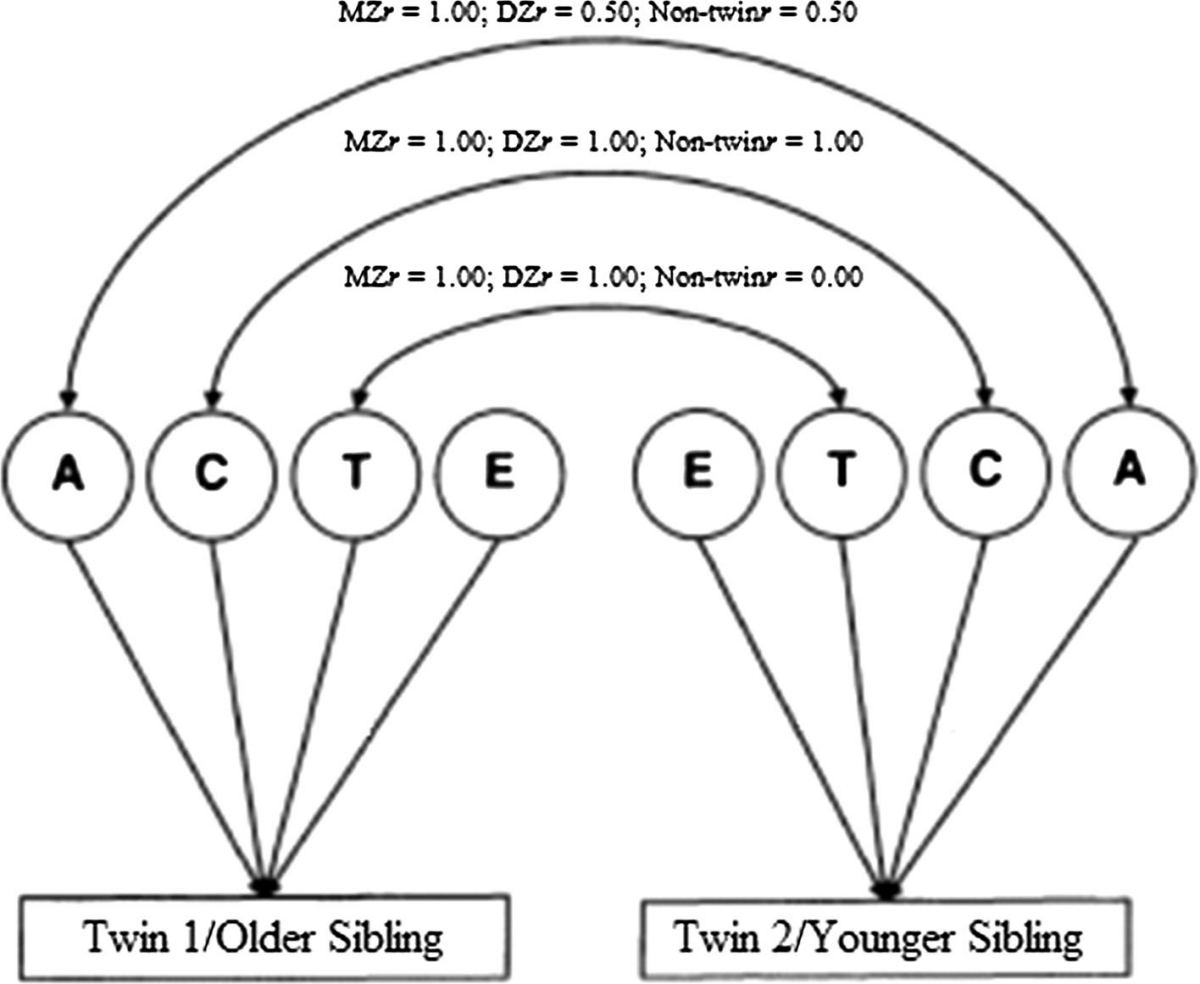
A univariate ACTE quantitative genetic model. Twin 1/Older Sibling and Twin 2/Younger Sibling are measured variables for the two twins/siblings—here, either mother reports of SRQ positivity, mother reports of SRQ negativity, father reports of SRQ positivity, or father reports of SRQ negativity, each for both twin 1/older sibling and twin 2/younger sibling. The latent variables A, C, T and E are the genetic factor, the shared environmental factor, the twin-specific environmental factor, and the non-shared environmental factor, respectively. The *curved*, *two-headed arrows* indicate correlations between the variables they connect; the *one-headed straight arrows* represent paths, standardised partial regressions of the measured variables on the latent variable

Figure 1 shows the basic structure of the model. It takes into account genetic theory at the level of the correlations between the latent variables (A, C, T, and E). The curved double-headed arrow linking ‘A’ between the siblings is set to 1.00 for MZ twins, because these pairs share 100 % of their genes, and 0.50 for DZ twins and non-twin siblings, because these pairs share 50 % of their genes. The path linking ‘C’ for the twins/non-twins is set to 1.00 in all cases, thereby equating the shared environment across all pairs, because siblings in all three groups were reared together. The arrow linking ‘T’ is set to 1.00 for both MZ twins and DZ twins, and 0.00 for non-twin sibling pairs, assessing the extent to which environmental factors that are specific to twins are important. ‘E’ for the three groups is not joined by a curved double-headed arrow, because non-shared environmental influences are not common to both siblings, and instead account for intra-pair differences not accounted for by non-shared genes. This component also contains measurement error.

We calculated four separate univariate ACTE models, for mother reports of SRQ positivity, mother reports of SRQ negativity, father reports of SRQ positivity, and father reports of SRQ negativity respectively. Table 6 contains the results of the univariate analyses. Significant heritability estimates emerged for mother reports of SRQ positivity, mother reports of SRQ negativity, and father reports of SRQ negativity, but not for father reports of SRQ positivity. Genetic influences explained a moderate proportion of variance in mother reports of SRQ positivity, whereas these values were modest for mother reports of SRQ negativity and father reports of SRQ negativity.

**Table 6:** Model fitting estimates of genetic and environmental components of variance for the MISR composite scales

MISR scales	Components of variance				AIC value
	h^2^	c^2^	t^2^	e^2^	
Mother reports SRQ positivity	0.49* [0.37–0.64]	0.40* [0.25–0.51]	0.00 [0.00–0.13]	0.11* [0.08–0.15]	1534.35
Mother reports SRQ negativity	0.34* [0.24–0.45]	0.33* [0.19–0.52]	0.25* [0.13–0.42]	0.08* [0.06–0.11]	1789.71
Father reports SRQ positivity	0.17 [0.00–0.32]	0.70* [0.51–0.81]	0.00 [0.00–0.20]	0.14* [0.09–0.21]	701.00
Father reports SRQ negativity	0.30* [0.19–0.44]	0.26* [0.11–0.47]	0.40* [0.15–0.58]	0.05* [0.03–0.08]	805.75

Values in parentheses are confidence intervals for each variance estimate

*MISR* maternal interview of sibling relationships, *SRQ* sibling relationship quality, *h* ^2^ additive genetic variance, *c* ^2^ shared environmental variance, *t* ^2^ twin-specific environmental variance, *e* ^2^ non-shared environmental variance, *AIC* akaike information criterion

* Confidence intervals indicate the value is significant

The shared environmental estimates accounted for the most variance in SRQ overall, and were moderate to substantial and significant across all four of the MISR measures. This component was particularly high for father reports of SRQ positivity. Negligible and non-significant estimates for the twin-specific environment were revealed for both mother and father reports of SRQ positivity. However, this estimate was modest for mother reports of SRQ negativity, and moderate for father reports of SRQ negativity. The non-shared environment accounted for small but significant amounts of variance for all four sibling relationship measures.

The findings from the univariate analyses partially supported our hypothesis (d), that SRQ positivity would yield modest genetic influence and substantial shared environmental influence, and our hypothesis (e), that SRQ negativity would yield substantial genetic influence and modest shared environmental influence. Genetic estimates were modest to moderate across both SRQ positivity and negativity, and non-twin specific environmental estimates were moderate to substantial.

## Discussion

We set out to compare the nature of the relationship between MZ twins, DZ twins, and non-twin siblings, and to disentangle genetic and environmental contributions to SRQ using these three sibling groups. Surprisingly, and in opposition to both inclusive fitness theory (Neyer and Lang 2003) and to our first hypothesis (a), MZ twin pairs within our sample did not have significantly higher levels of SRQ positivity, or significantly lower levels of SRQ negativity, than did DZ twin pairs or non-twin pairs. Likewise, the derivative of secure attachment theory put forward by Tancredy and Fraley (2006), informing our second hypothesis (b), was not supported-MZ twins and DZ twins did not have significantly higher levels of SRQ positivity, or significantly lower levels of SRQ negativity, than did non-twin siblings. In other words, there were no substantial differences in the ways that siblings of varying genetic relatedness behaved towards one another. In relation to our third hypothesis (c), sex constellation differences did emerge, partially supporting the prediction proposed. Our fourth and fifth hypotheses (d) and (e) were also partly verified, through the modest to moderate genetic influence and the moderate to substantial shared environmental influence for SRQ. Note, however, that the only significant difference found between SRQ positivity and SRQ negativity was when the twin-specific environment was considered.

### Theoretical explanations of SRQ

The current study did not support Neyer and Lang’s (2003) proposal, that MZ twins have a special regard for one another because they share more of their genetic makeup than do DZ twins or non-twins. Within our sample, parents reported that MZ twins behaved no more positively, and no less negatively, towards each other than did DZ twins or non-twin siblings. Consequently, kinship theory cannot be applied here, because there was no evidence that identical twins were more motivated to behave altruistically towards each other. The measures employed in our study did differ from those of previous papers in important ways, and thus may have caused this discrepancy in findings. Indeed, we did not ask parents to directly rate levels of siblings’ closeness or dependence, as Neyer and Lang (2003), Neyer (2002) and Penninkilampi-Kerola et al. (2005) did. Rather, we primarily asked mothers and fathers about each child’s observable actions towards his/her sibling, making the questionnaire less driven by subjective perceptions, and more focused on actual behaviour.

Correspondingly, the nature of SRQ across twins (in general) and non-twins was similar. This finding also disconfirms the alternative theoretical perspective regarding twin attachments put forward by Tancredy and Fraley (2006)—that all twins (whether MZ or DZ) form distinctively close relationships in comparison to non-twin brothers and sisters. When reviewing these results, we must take into account the young age, and small age range, of our samples, compared to those in the previous studies (Tancredy and Fraley 2006; Fraley and Tancredy 2012). During this early developmental stage, children are typically living at home and sibling relationships are particularly intense, with brothers and sisters often spending more time with one another than with their parents (McHale and Crouter 1996). It may be fair to say then, that these ties during early childhood are relatively ‘forced’-individuals making up sibling dyads have little choice but to engage with each other. Because of this, a measure of SRQ at this age may not be indicative of long-term relational characteristics that differentiate between twin versus non-twin siblings. Perhaps the enduring nature of SRQ attachments only becomes clear when children are older, spending more time away from the family home and being able to choose the extent of time and effort they put into their sibling relations (Furman and Buhrmester 1992).

### Sex constellation differences in SRQ

Differences did emerge in relation to gender. Specifically, higher levels of SRQ negativity were found for male pairs than for both female pairs and opposite-sex pairs. However, this finding only held true for mother reports. Inspection of the means showed that mothers reported higher levels of negativity between boy-boy twins than did fathers, perhaps because the mothers in these relatively traditional samples spent more time with the children, providing them with more opportunities to witness the more frequent negative behaviours. It was also hypothesised that female-female sibling dyads would have higher levels of SRQ positivity than male-male or opposite-sex dyads, yet this was not confirmed. The general pattern of results in relation to negativity within this sample was consistent with our expectations, and runs parallel to Brody et al. (1985) and Maccoby’s (1998) earlier gender work. It also ties in with Nash’s (1979) notion that caregivers tend to teach boys the meaning of social relationships to a lesser extent than to girls. Saying that, it was surprising that the sex constellation of dyads did not play a part in SRQ positivity, as Abramovitch et al. (1979, 1986) and Buist et al. (2002) have reported that cooperation between siblings also varies by gender.

### BG and SRQ

The MZ twins were rated as more similar in their behaviour towards one another than were the DZ twins, leading to modest to moderate heritability estimates. Given that these sibling behaviours were based on parent reports, it could be that this merely reflected parents’ *beliefs* about their twins. However, our finding of genetic influence confirms previous work reviewed by McGuire et al. (2015), bolstering our interpretation that genes themselves are a likely causal agent. The effect of genes on the bond between siblings is suggestive of the children’s own characteristics contributing to SRQ, and indeed the existing evidence endorses this. For example, Lemery and Goldsmith (2001) and Pike and Atzaba-Poria (2003), who also used twin pairs within their study design, found that the target children’s temperaments could explain much of the moderate genetic influence they uncovered. Lemery and Goldsmith (2001) discovered this result when using parental reports of sibling conflict in their young children, and Pike and Atzaba-Poria (2003) concurred when they gathered teenagers’ reports of their own hostility and rivalry towards their co-twin.

Also in line with the existing evidence (Lemery and Goldsmith 2001; McGuire et al. 2000; Reiss et al. 2000; Rende et al. 1992), parents tended to rate their children’s behaviour towards one another in similar terms, yielding a large shared environmental component. This is indicative of consistency among children within the same family, once genetic similarity has been accounted for. To the extent that such a result is reflective of true behavioural similarity, it is congruent with Bowen’s (1978) family systems theory. Siblings and twins growing up within a family can have similar experiences, and the characteristics of specific dyadic bonds can ‘spill over’ and influence other familial interactions (Engfer 1988). Lemery and Goldsmith have argued that the parent–child relationship, and parenting per se, is the most salient aspect of the shared environmental contribution to relationships between siblings. As well as the individual impact of mothers’ and fathers’ parenting, siblings may be exposed to other collective familial elements that can lead to high shared environmental estimates. For example, parental mental health, marital quality, and socioeconomic status are all family-wide factors, which may act to make siblings more similar to one another (Rowe 1983).

We were also able to investigate the extent to which environmental factors that were specific to twins influenced variation in SRQ. Interestingly, this twin-specific effect was moderate and significant for negativity, but not positivity. This indicates that parents of both MZ and DZ twins reported their children demonstrating more similar levels of conflictual behaviour than did parents of non-twin siblings. We propose two possible interpretations for such a finding. It may be that mothers and fathers of twins *perceived* these pairs to be more reciprocal in their negative interactions, and thus overestimated behavioural similarities between them in their SRQ reports. Alternatively, the parental accounts documented here could have reflected reality—it may be that twin dyads genuinely were more reciprocal in their negative sibling behaviours than were non-twins.

### Limitations and future directions

We used parental self-report measures to assess the quality of sibling relationships within our sample. However, parents tend to overestimate the consistency of both their behaviour towards their offspring, and of their children’s behaviour towards them and other family members (Pike et al. 1996). A future study may therefore benefit from using videotaped sibling interactions, which would allow SRQ to be rated by trained researchers during a standardised semi-structured task. Saying this, we would argue that all methodologies have their flaws (Pike and Oliver 2015), and several perspectives are needed to capture the intricacies of the sibling bond.

In addition, there were relatively low levels of negative SRQ and relatively high levels of positive SRQ in both our twin and non-twin samples. While families were broad in educational qualifications and occupational status in both studies, neither sample was fully representative of the UK population, particularly at the lower end of the socioeconomic spectrum. Numerous studies have found that factors such as social class or race can affect family dynamics (Bronfenbrenner 1979), and sibling interactions can be intertwined with other familial relationships (Engfer 1988). Further explorations into SRQ are needed within more diverse samples, including broadening ethnicity, family type, and socioeconomic status, to determine whether our findings can be generalised across these groups.

## Conclusions

Importantly, the current research demonstrates that studies of twins in childhood can be generalised to the wider non-twin sibling population. We discovered no differences in the quality of the bond between identical twin pairs, fraternal twin pairs, and non-twin sibling pairs. In other words, these distinct groups of siblings behaved similarly to each other, displaying equivalent levels of positivity and negativity within their relationships. This was an unexpected finding, and one that has repercussions for the broader application of results from future twin-based investigations. We also conclude that genetically-influenced traits of children impact upon their sibling interactions, but that, unlike other family relationships, sibling dynamics are primarily characterised by reciprocity.
